# Does compulsory community treatment for discharged forensic hospital patients work? The recent evidence base

**DOI:** 10.1017/S1092852925000306

**Published:** 2025-06-13

**Authors:** Melinda DiCiro, Melanie Scott, Sean Sterling

**Affiliations:** 1 Forensic Services Division, California Department of State Hospitals, Sacramento, CA, USA; 2 Community Forensic Partnerships Division, California Department of State Hospitals, Sacramento, CA, USA

**Keywords:** Forensic psychiatry, community treatment, recidivism, Conditional Release Programs (CONREP), criminal justice, public safety

## Abstract

**Objective:**

Over the decades, research has demonstrated that Conditional Release Programs (CONREP) and Compulsory community treatment can reduce recidivism among forensic patients discharged from inpatient commitment. This study synthesizes current knowledge—including findings from a 2024 California Department of State Hospitals report—to evaluate the impact of involuntary community treatment on recidivism and patient outcomes.

**Methods:**

We retrospectively analyzed 2613 patients discharged from California state hospitals between 2012 and 2017. Patients were either directly discharged to the community (N = 2011) or referred to CONREP (N = 602). Data on rearrests for general and violent offenses were obtained through 2018. Variables with established relationships to recidivism (e.g., commitment category, mental health diagnoses, lengths of stay) were included. Statistical analyses, including chi-square tests, Cox regression, and logistic regression, were conducted to compare recidivism rates and identify significant predictors.

**Results:**

CONREP-treated patients demonstrated dramatically lower fixed recidivism rates at 1, 3, and 5 years compared with directly discharged patients. Direct discharge was associated with up to a sevenfold increased likelihood of rearrest within 1 year. The median time to rearrest was 400 days for directly discharged patients versus 500 days for CONREP patients (*p* < .004). Logistic regression revealed that direct discharge, younger age, and a higher number of state hospital commitments were significant predictors of rearrest.

**Conclusions:**

Structured, court-supervised community treatment via CONREP substantially reduces recidivism among forensic patients, promoting safer community reintegration and improved outcomes. These findings support expanding CONREP services to enhance public safety and patient rehabilitation.

## Introduction

Over the decades, research has demonstrated the efficacy of Conditional Release Programs (CONREP) and Compulsory community treatment for forensic patients following discharge from inpatient commitment. Since a forensic commitment is based on criminal behavior, the primary concern is the risk of criminal and violent behavior. While secure forensic hospitalization can constrain criminal and violent behavior, there are drawbacks, including the criminalization of mental illness, high costs, and undermining patient autonomy and functioning. Furthermore, the principles of treatment in the least restrictive alternative (*Olmstead v. LC*, 1999) and Risk, Need, Responsivity (RNR) support community treatment when feasible and effective. Therefore, continued study of these programs and their utility is essential for establishing best practices.

Additionally, understanding the factors influencing recidivism for patients released from forensic hospitals informs policies necessary for public safety, while reducing the criminalization of mental illness. The growing focus on community treatment for forensic populations highlights the need to identify factors contributing to success. As such, this article synthesizes current knowledge on Compulsory community treatment’s impact on recidivism among discharged forensic patients, highlighting its role in improved outcomes and safety for individuals and the community. In doing so, we review findings from a 2024 California Department of State Hospitals (DSH) report, from our perspectives as study researchers (Drs. DiCiro and Sterling) and as an overseer of CONREP (Dr. Scott).

### CONREP and Compulsory court supervision

Depending on the jurisdiction, CONREP and Compulsory court supervision are mechanisms wherein a court or administrative law hearing can mandate outpatient treatment and supervision for forensic patients released from a forensic hospital. The goals of these programs are to (1) provide necessary treatment and support for those with severe and persistent mental health disorders, (2) ensure a successful transition to the community, and (3) mitigate potential dangerousness and protect public safety.^1^ ^,^^2^

Most CONREP and similar programs include housing support, case management, individual and group therapy, medication management, substance use screening and treatment, home visits, and other services tailored to individual needs and risk levels. When necessary, CONREP programs can temporarily rehospitalize a patient who decompensates, shows signs of dangerousness, or violates the terms and conditions of release. In addition, a court can revoke CONREP treatment for unsuccessful programming or committing a new offense. Supervised outpatient treatment through CONREP has consistently been shown to effectively reduce criminal recidivism and improve forensic patient outcomes in the United States and internationally.

### Recidivism rates for direct hospital discharges

The literature has consistently demonstrated that patients unconditionally released or directly discharged from forensic hospitals evidence poor treatment outcomes and significantly higher rates of recidivism. Such outcomes were demonstrated in a study by Fazel et al.,^3^ which analyzed 6520 patients discharged from psychiatric hospitals in Sweden between 1973 and 2009, wherein 69% were rehospitalized, and 40% were convicted for a violent offense after discharge. Likewise, Wolf et al.^4^ followed 2248 patients directly discharged from forensic hospitals in Sweden between 1992 and 2013 and found that 6.9% violently recidivated within 12 months and 10.9% within 24 months; the strongest predictors of violent recidivism were previous violent crime and male gender. They also noted that longer lengths of stay reduced recidivism for those tied to Compulsory outpatient treatment after hospital discharge.

Siddiqui et al.^5^ examined post-discharge recidivism among 66 patients directly discharged from a forensic psychiatric service in Saudi Arabia between 2005 and 2020. Results found that 68.18% were rehospitalized during the 15-year study period, and 16.66% violently offended after discharge. Ojansuu^6^ found similar results in exploring criminal and violent recidivism among 501 patients directly discharged from forensic hospitals in Finland between 1999 and 2018. On average, patients spent 10 years committed within the forensic hospital, and 16.6% re-offended after discharge (mean time to conviction of 3.8 years). Furthermore, the re-offense was considered violent in 9.6% of the sample. Sample characteristics showed that the patients were primarily diagnosed with schizophrenia (91%) and that more than half had a co-occurring substance use disorder (63.5%). Additionally, a longer treatment duration was associated with a reduced likelihood of general recidivism.

### Recidivism rates for discharge to CONREP

Overwhelmingly, the literature has also shown that patients discharged from a forensic hospital to CONREP consistently have lower recidivism rates than those directly discharged from forensic hospitals without CONREP treatment. Reynolds^7^ highlights this using a sample of 110 patients released into CONREP from the Missouri Department of Mental Health. Results showed only a 1% recidivism rate and a 7% revocation rate after a 3-year follow-up. Similarly, Parker^8^ found that for an Assertive Community Treatment program that supervised 83 Not Guilty by Reason of Insanity (NGRI) patients in CONREP, after a 5-year follow-up, only 5 of the 83 patients were rearrested. This pattern of low recidivism rates was also found at the Oregon CONREP, where, in a sample of 238 patients with NGRI, recidivism rates were less than 1%, with only moderate revocation rates (*n* = 81, 33.6%).^9^

In a California study by McDermott et al.,^1^ the authors tracked 93 NGRI patients discharged from a California state hospital (2002–2013) for 4.83 years, comparing those unconditionally discharged directly from the state hospital with those discharged to CONREP treatment in the community. Results found that nearly half of those unconditionally discharged (43.8%) were rearrested during the study period compared to those released under CONREP (8.2%). Additionally, those who were NGRI but restored to sanity and unconditionally released also had higher arrest rates (25%). While time to rearrest did not differ among groups, those released to the community without CONREP were nearly nine times more likely to re-offend than those released with CONREP. Patient characteristics showed that CONREP patients were more often White, with a diagnosis of schizophrenia, while those unconditionally discharged were often diagnosed with a personality disorder. Overall, the results showed that court-mandated treatment (CONREP) had the most potent effect on preventing arrests.

From 2008 to 2015, Rossetto et al.^10^ examined female forensic patients rehospitalized after conditional or unconditional discharge from Italian Residences for the Execution of the Security Measure (REMs)—small, local treatment centers that replaced forensic hospitals and are integrated with community psychiatry resources. Follow-up was conducted through 2018. Overall, the results found that for patients conditionally released, 70.8% did not require readmission or rehospitalization. Key predictors of rehospitalization were having a substance use disorder, having a personality disorder, being unconditionally discharged, younger age, and having a shorter inpatient stay. The diagnosis of schizophrenia was unrelated. The authors posited that readmission most depended on whether the person was discharged on conditional release or not.

Also, in a Swedish study, Noland and Strandh^11^ used a survival analysis to examine 1150 forensic patients discharged from forensic hospitals between 2009 and 2018. Outcomes showed that older age at discharge was associated with a lower likelihood of recidivism, while having a substance use disorder, a history of crime before the index offense, or a diagnosed personality disorder without psychosis was associated with a higher likelihood of recidivism.

### Treatment outcomes without CONREP or Compulsory outpatient treatment

Not all states utilize CONREP or Compulsory outpatient treatment upon discharge from forensic hospitals. For example, in England, Westhead et al.^12^followed a group of 843 patients—70.3% of whom were admitted from prison to a medium secure psychiatric facility between July 1983 and June 2013. The patients were discharged to prisons, high, medium, low, and nonsecure hospital facilities, as well as the community. Of the 843 discharged patients, 43.8% were convicted of a new offense within the first 5 years of release. The conviction rate for “serious offenses” (defined as offenses warranting a life sentence) was 3.1%. Additionally, 61.6% of the patients were readmitted to a psychiatric inpatient service (though not necessarily to a forensic hospital). There was also a higher long-term risk of premature mortality. Of those released directly to the community, two-thirds were readmitted to a psychiatric service, with 31.8% readmitted within the first year. The authors emphasized the need for treatment and supervision after hospital discharge to assist in reintegration.

Similarly, in examining release without CONREP, Haroon et al.^13^ examined post-release outcomes of insanity acquittees discharged from state hospitals in North Carolina between 1996 and 2020. North Carolina is unique because it is one of eight states that lack an enforceable court monitoring program for released state hospital patients. The follow-up period ranged from 1 year to almost 23 years. Overall, the authors assert that conditionally released patients have lower rates of rearrest and rehospitalization compared to those unconditionally discharged. Specifically, the median time to rehospitalization for unconditionally released patients was 1.8 years, and 27.9% of acquittees were rehospitalized after unconditional release. Additionally, 14.8% had re-offended and were convicted of a new crime within a median period of 2 years. Furthermore, the authors compared recidivism in North Carolina to that of different states using CONREP. They concluded that insanity acquittees in North Carolina have higher rates of criminal recidivism than acquittees in other states due to lack of CONREP.

Reynolds^14^ provided a commentary on the study by Haroon et al.^13^ and championed the value of CONREP. He noted that the reconviction rate in the Haroon et al. study parallels that of unsupervised (ie, directly discharged) forensic patients in other states but substantially exceeds that of court-supervised CONREP. Citing his experience in the Missouri and Colorado systems, Reynolds agreed with Haroon et al. that the absence of court supervision accounts for higher recidivism rates. Reynolds highlighted the benefits of court authority to monitor patients and address conditional release violations through early detection and intervention, thereby reducing unnecessarily long hospitalizations and supporting better outcomes while lowering costs.

Finally, regarding sexually violent persons on supervised release, Ambroziak et al.^15^ explored re-offense recidivism for 205 sexually violent persons on supervised release from a state forensic hospital over the years. Although this patient population did not evidence high rates of severe mental illness compared with other post-release recidivism populations, the results revealed the value of community supervision. The authors found a 1.5% rate of new sex offense charges in the supervised release sample, whereas a comparable group of offenders released directly from prison to the community (with fewer restrictions and less oversight) recidivated at nearly double that rate (2.9%), despite a more conservative outcome variable (convictions).

### The 2024 California DSH CONREP effectiveness study

In California, CONREP programs provide oversight, structure, and treatment and serve as a step-down for reintegration into the community. The statute includes a mechanism for rehospitalization when patients show signs of decompensation or violate the conditions of their release. A court can also revoke the CONREP status. Patients are released to CONREP when a court deems them able to meet the program’s terms and conditions and can be treated safely and effectively in the community. Alternatively, patients may be directly discharged from the hospital to the community when a court finds they no longer meet commitment criteria. Comparing CONREP-treated and directly discharged patients reveals the value of ongoing, court-supervised treatment for forensic patients.

CONREP comprises patients from the following five primary commitment schemes: Not Guilty by Reason of Insanity (NGI, also known as insanity acquittees); Incompetent to Stand Trial (IST); Offenders with Mental Health Disorders (OMD-Parole), a postprison civil commitment scheme as a condition of parole for those who remain dangerous due to their mental disorder; Offenders with Mental Health Disorders (OMD-Civil), a civil commitment scheme for those who remain dangerous beyond the parole period; and Sexually Violent Predator (SVP), a postprison civil commitment scheme for individuals whose mental disorders render them predisposed to commit predatory sex offenses.

Between 1990 and 2002, the California DSH conducted five evaluations of its CONREP, consistently demonstrating the program’s effectiveness in reducing recidivism. The initial 1990 study of 710 patients showed that CONREP participants had significantly lower rearrest rates than those directly discharged patients (6.7% vs. 27.3%). Subsequent studies in 1993, 1998, 1999, and 2002 confirmed these positive outcomes, with the 2002 analysis of 2101 patients showing an overall rearrest rate of 8.9% for CONREP participants. Throughout these studies, CONREP patients demonstrated improved social functioning and lower recidivism rates across all commitment categories, although rates varied by legal classification. The program’s success was attributed to enhanced patient functioning during treatment and the ability to temporarily rehospitalize struggling patients. Notably, CONREP consistently proved to be more cost-effective, operating at approximately 20% of the state hospital’s costs while maintaining better outcomes.

Following a hiatus after the 2002 report, a July 2024 report confirmed earlier California findings, reinforcing CONREP’s role in enhancing patient outcomes and public safety. The 2024 California DSH CONREP Effectiveness Study compared outcomes between patients directly discharged from state hospitals and those released through CONREP (CONREP-treated). This report provides compelling evidence for the program’s effectiveness in patient treatment, reintegration, and public safety. As the primary researchers for this study, we summarize the most salient outcomes and conclusions, augmenting our summary with statistical analyses and details not explicated in the government report.

## Methods

We analyzed rearrests for general and violent crime and other relevant variables for 2613 patients who were either discharged directly to the community (*N* = 2011) or discharged to CONREP (*N* = 602) from California state hospitals between 2012 and 2017. Follow-up through 2018 permitted at least 1 year in the community for all patients studied. The study population included individuals in four of the five commitment categories described above. This study used the first identified arrest event as the recidivism outcome variable. We chose available variables with established relationships to recidivism and violence in populations with severe mental illness (Bonta et al.,^16^ Harris et al.,^17^ among others), including commitment category, mental health diagnoses, and lengths of stay. We also examined the time of arrest.

Data were collected through the California DSH and CONREP tracking systems, linked with California Department of Justice (DOJ) arrest and prosecution records. The project was approved by the California Committee for the Protection of Human Subjects, which waived informed consent due to the retrospective, data-based nature of the study. We followed DSH deidentification protocols, and the results were deemed to be at low risk of identifying individual patients. Statistical analyses were performed using SPSS Version 23.

## Results

**Figure 1.**
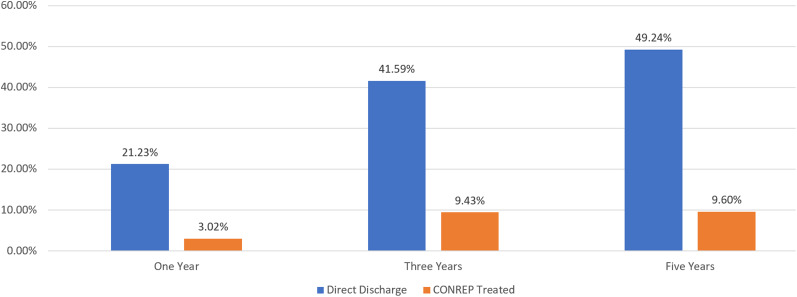
Fixed recidivism rates by treatment status. Reprinted from the California Department of State Hospitals CONREP Effectiveness Study (2024).

The study revealed dramatically lower fixed recidivism rates for any arrest among CONREP-treated patients compared to directly discharged patients across 1-, 3-, and 5-year time frames. Directly discharged patients were seven times more likely to be rearrested within 1 year and four and a half times more likely within 3 years. CONREP-treated patients maintained a significantly lower probability of rearrest at 5 years (five times lower). Note: Known deaths were removed from the calculation for the corresponding interval. The DSH CONREP Effectiveness Study (2024) also examined rearrests for sex offenses as well as other variables not discussed in this article. Statistical analyses (using chi-square tests on 2 × 2 contingency tables) revealed significant differences in recidivism rates, as shown in Figure 1 and Tables 1–3.

**Table 1.** One-year fixed recidivism for any arrest

	Direct discharge	CONREP treated
*N* = with at least 365 days (1 year) post discharge	2003	596
*N* = arrested within 365 days	427	18
Recidivism rate	21.23%	3.02%

*Note*: Odds ratio 8.70; .95 CI 5.37 to 4.07; Phi = +.2 *χ* ^2^ Pearson 109.38; *p* < .0001.

**Table 2.** Three-year fixed recidivism for any arrest

	Direct discharge	CONREP treated
*N* = with at least 1095 days (3 years) post discharge	1207	350
N = arrested within 1095 days for this group	502	33
Recidivism rate	41.59%	9.43%

*Note*: Odds ratio 6.84; .95 CI 4.69 to 9.96; Phi = +.28 *χ* ^2^ Pearson 124.44; *p* < .0001.

**Table 3.** Five-year fixed recidivism for any arrest

	Direct discharge	CONREP treated
*N* = with at least 1825 days (5 years) post discharge	463	125
*N* = arrested within 1825 days for this group	228	12
Recidivism rate	49.24%	9.60%

*Note*: Odds ratio 9.13; .95 CI 4.90 to 17.02; Phi = +33 *χ* ^2^ Pearson 64.04; *p* < .0001.

### Violent recidivism

**Figure 2.**
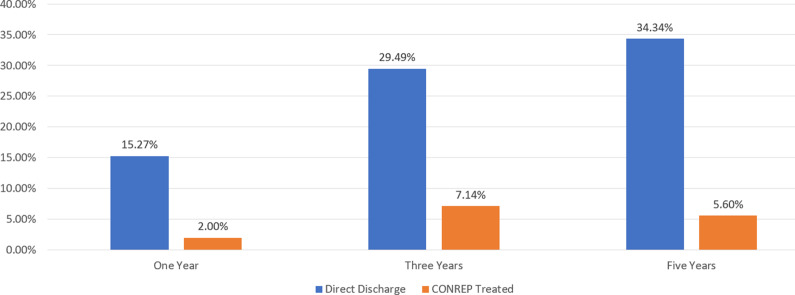
Fixed recidivism rate for violent offenses by treatment status. Reprinted from the California Department of State Hospitals CONREP Effectiveness Study (2024).

Results revealed dramatically lower fixed recidivism rates for violent arrests among CONREP-treated patients compared with directly discharged patients across 1-, 3-, and 5-year timeframes. Violent offenses were defined as those leading to or posing a threat of physical injury or death, contact sex offenses, and actual or implied threats of violence. Directly discharged patients were nearly nine times more likely to be rearrested for a violent offense within 1 year, five times more likely within 3 years, and almost nine times more likely within 5 years. As shown in Figure 2 and Tables 4–6, statistical analysis shows significant differences in violent recidivism rates.

Tables 4–6 compare fixed recidivism rates for violent offense arrests.

**Table 4.** One-year fixed recidivism rates for a violent offense

	Direct discharge	CONREP treated
*N* = with at least 365 days (1 year) post discharge	2003	596
*N* = arrested within 365 days	306	12
Violent recidivism rate	15.27%	2.0%

*Note*: Odds ratio 8.77; .95 CI 4.89 to 15.74; Phi = + .17, *χ* ^2^ Pearson 75.25; p < .0001.

**Table 5.** Three-year fixed recidivism rates for a violent offense

	Direct discharge	CONREP treated
*N* = with at least 1095 days (3 years) post discharge	1207	350
*N* = arrested within 1095 days for this group	356	25
Violent recidivism rate	29.49%	7.14%

*Note*: Odds ratio 5.43; .95 CI 3.55 to 8.32; Phi = +.22, *χ* ^2^ Pearson 73.34; *p* < .0001.

**Table 6.** Five-year fixed recidivism rates for a violent offense

	Direct discharge	CONREP treated
*N* = with at least 1825 days (5 years) post discharge	463	125
*N* = arrested within 1825 days for this group	159	<11^a^
Violent recidivism rate	34.34%	~6.0%^b^

*Note*: Odds ratio 8.81; .95 CI is 4.01 to 19.35; Phi = +.26 *χ* ^2^ Pearson 40.13; *p* < .0001. *N* arrested within 1825 days: direct discharge = 159; CONREP treated = 7. Violent recidivism rate: direct discharge = 34.34%; CONREP treated = 5.60%.Odds ratio: 8.81; 95% CI 4.01 to 19.35; Phi = +.26; *χ* ^2^ Pearson = 40.13; *p* < .0001.

a Numbers <11 not published for data deidentification.

b Approximate percentage for deidentification.

A Cox regression analysis determined that, for those arrested, half of the CONREP-treated patients were arrested at approximately 500 days versus approximately 400 days for directly discharged patients (*χ* ^2^ = 10.942, df = 2, *p* < .004). Further analysis revealed that three-quarters of the arrested CONREP-treated patients were arrested within the first 18 months. Longer lengths of stay in CONREP correlated with lower recidivism (point biserial correlation; Pearson *r* = −.082; *p* = .026, one-tailed test).

We also found distinct differences between CONREP-treated patients and those directly discharged. CONREP participants tended to be older (mean age 45.48 vs. 42.11 years), had more extended hospital stays (mean 1895.67 vs. 797.7 days), and included more female patients (23.8% vs. 5.1%). Additionally, CONREP patients were more likely to have psychotic disorders and less likely to have previous state hospital commitments or personality disorders. Because the literature consistently associates these factors with lower recidivism, these group differences likely partially account for their lower recidivism rates. Table 7 shows the differences between the groups.

**Table 7.** Group differences between directly discharged and CONREP-treated patients

Variable	Group difference and direction	Significance
Gender	Those in the directly discharged group were **more** likely to be **male.**	Odds ratio 5.98; Phi +.27 *χ* ^2^ Pearson 192.02 *p* < .0001
Age at discharge	The CONREP treated group was **older** than the directly discharged groups.	An independent samples *t*-test Results: *t* = 6.050 (df) = 990.944; *p* = .000 two-tailed test.
Race-ethnicity	Those in the directly discharged group were	
	**Less** likely to be White	Odds ratio .68; Phi −.08 *χ* ^2^ Pearson 17.41 *p* < .0001
	**More** likely to be Black.	Odds ratio 1.31 Phi+.05 *χ* ^2^ Pearson 6.11 p < .01
	**More l**ikely to be Hispanic.	Odds ratio 1.31 Phi+.05 *χ* ^2^ Pearson 6.11 *p* < .01
	**Less** likely to be Asian	Odds ratio .55 Phi+.06 *χ* ^2^ Pearson 8.58 *p* < .003
Hospital LOS	CONREP treated patients had **longer** lengths of stay in the hospital than did directly discharged patients.	Independent samples *t*-test: *t* = 16.274 (df) =775.53; *p* = <.000 two-tailed test.
Commitment offense category	Directly discharged patients were less likely to have a violent commitment offense.	Odds ratio .48; Phi −.11 *χ* ^2^ Pearson 32.8 *p* < .0001
	Directly discharged patients were significantly more likely to have a sex offense.	Odds ratio 2.64 Phi +.1 *χ* ^2^ Pearson 25.5 *p* < .0001
Commitment offense severity	The mean commitment offense severity for the CONREP treated group was **higher** than that of the directly discharged groups.	An independent samples *t*-test: *t* = 8.3426. (df) =975.788; *p* < .000 two-tailed test.
Commitment type	Directly discharged patients **were** more likely to be OMD parole.	Odds ratio 19.11 Phi −.51 *χ* ^2^ Pearson 1. 96 688 *p* < .0001
	**Less** likely to be OMD civil.	Odds ratio .68 Phi −.06 *χ* ^2^ Pearson 9.69 *p* < .0001
	**Less** likely to be NGI	Odds ratio .05 Phi −.59 *χ* ^2^ Pearson 896.3 *p* < .0001 Odds ratio 21.32 Phi −.59 *χ* ^2^ Pearson 912. 94 *p* < .0001
	**More** likely to be SVP.	Odds ratio 2.83 Phi −06 *χ* ^2^ Pearson 9.81 *p* < .0001
Number commitments		Odds ratio 5.1 Phi +.06 *χ* ^2^ Pearson 71.3 *p* < .0001
Diagnostic category	Directly discharged patients were **less** likely to have a psychotic disorder.	Odds ratio .60 Phi −.1 *χ* ^2^ Pearson 223.7 *p* < .0001
	**More** likely to have a paraphilic disorder.	Odds ratio 3.4 Phi +.11 *χ* ^2^ Pearson 24.57 *p* < .0001
	**More** likely to have a depressive disorder.	Odds ratio 1.53 Phi +.04 *χ* ^2^ Pearson 3.61 *p* < .057
	About **equally** likely to have a substance use disorder	Odds ratio .91 Phi −.002 *χ* ^2^ Pearson .76 *p* = .38 ns
	**More** likely to have ASPD	OR 3.27 Phi +.09 *χ* ^2^ Pearson 19.5 *p* < .0001

We conducted a logistic regression analysis of potentially impactful variables to examine the impact of factors affecting rearrest. The most potent contributor to rearrest was being directly discharged, followed by a younger age, having more state hospital commitments, lower commitment offense severity, OMD commitment, and male gender. Ethnicity, psychotic disorder diagnosis, and personality disorder diagnosis were not significantly related to rearrest. Hospital LOS approached significance. The logistic regression model showed a significant association between variables and recidivism (shared variance of 22%), with good specificity (83% correctly classified as non-recidivating) but poor sensitivity (41% correctly classified as recidivating), resulting in an overall correct classification rate of 71%. Table 8 displays the logistic regression model.

**Table 8.** Logistic regression: factors impacting rearrest

Observed			Predicted		
			Recidivism		Percentage correct
			No	Yes	
Step 1	Recidivism	No	1388	287	82.9
		Yes	548	378	40.8
	Overall percentage				67.9
a. The cut value is .500					

Variables in the equation									
		B	S.E.	Wald	df	Sig.	Exp(B)	95% CI for EXP(B)	
								Lower	Upper
Step 1	Age at discharge	−.025	.004	35.554	1	.000	.975	.967	.983
	Gender	.397	.191	4.334	1	.037	1.488	1.094	2.163
	Length of stay in hospital	.000	.000	3.277	1	.070	1.000	1.000	1.000
	Number of commitments	.637	.110	33.855	1	.000	1.891	1.528	2.344
	OMD_in	.732	.154	22.605	1	.000	2.080	1.538	2.812
	Any personality disorder	.110	.162	.457	1	.499	1.116	.812	1.533
	Any psychotic disorder	−.045	.104	.193	1	.661	0.956	.78	1.171
	Commitment at minima severity	−0.116	.024	24.373	1	000	0.890	.85	.932
	CONCEPT treated	−1.118	.161	47.967	1	000	0.327	.239	.449
	Work ethic	−.46	.093	.247	1	.619	0.955	.798	1.145
	Constant	−.281	.540	.270	1	.603	0.755		

### Duration of treatment effectiveness

The research demonstrated that CONREP’s impact extended beyond the active treatment phase. Only 4% of the patients were arrested during treatment and only 7% recidivated after program completion. Notably, longer CONREP stays were correlated with better outcomes, suggesting a dose–response relationship between treatment duration and success.

### Comparison to a similar California population

**Figure 3.**
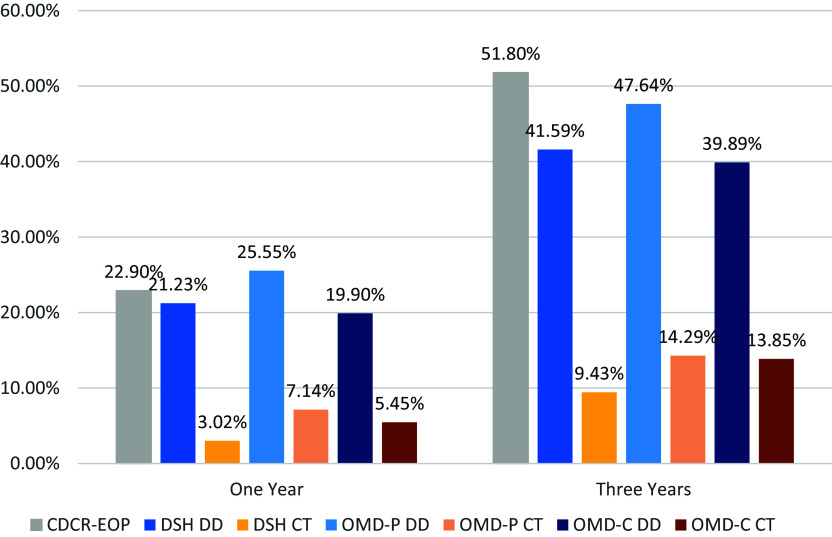
Reconviction rates for CDCR EOP prison releases and rearrest rates for OMD-P and OMD-C commitment DSH direct discharge and CONREP treated patients.

Because rearrest rates for OMD commitment categories (those committed to a state hospital after serving a prison sentence) were higher than those for NGI (those committed directly to a state hospital in lieu of a conviction and prison term), we compared the OMD rates to recidivism in an analogous population. Specifically, we compared the rearrest rates of directly discharged and CONREP-treated groups for the entire sample—and the OMD-Parole and OMD-Civil categories—to the reconviction rates of individuals from the California Department of Corrections and Rehabilitation (CDCR) who were enrolled in the Enhanced Outpatient Program (EOP) upon prison release (EOP is an intensive outpatient psychiatric treatment program in California Prisons). CDCR EOP reconviction rates were 22.90% at 1 year and 51.80% at 3 years. In comparison, rearrest rates in this study were 21.32% for directly discharged and 3.02% for CONREP-treated patients at 1 year, and 41.59% versus 9.43% at 3 years.^19^ For the OMD-Parole group, directly discharged patients had rearrest rates of 25.55% (1 year) and 47.64% (3 years), while CONREP-treated patients had rates of 7.14% (1 year) and 14.29% (3 years). For the OMD-Civil group, directly discharged rates were 19.19% (1 year) and 39.89% (3 years) versus 5.45% (1 year) and 13.85% (3 years) for CONREP-treated patients. Reconviction rates for the CDCR EOP group were consistently higher than the rearrest rates from the state hospitals, aside from a slightly higher rearrest rate for the OMD-Parole group after 1 year. Actual recidivism differences between the CDCR and DSH groups are likely higher, given that arrest is a more sensitive outcome variable than conviction. Figure 3 visually displays these differences.

These outcomes suggest that Compulsory treatment post-release may reduce recidivism for incarcerated individuals with severe mental health disorders, although further exploration is warranted, given potential confounding factors. Furthermore, before 2024, a high number of OMD-Parole patients were decertified by the Superior Court and released from the state hospital directly into the community within 5 days, leaving little time for care coordination. This gap may have contributed to the comparatively high rate for the OMD-Parole commitment category. Since 2024, the state hospital has been permitted up to 30 days to coordinate release plans, which could reduce the risk of recidivism.

Figure 3 visually displays these differences.

## Discussion

The results show that CONREP’s approach to graduated community reintegration of forensic patients provides substantial benefits. CONREP has the legal authority to ensure treatment compliance, including medication compliance and the required monitoring and support. Even though group differences complicate isolating CONREP-specific effects, the magnitude of the differences in outcomes strongly supports the conclusion that CONREP treatment contributes to reduced criminal and violent recidivism for forensic patients with severe mental illnesses. Consistent positive outcomes across groups—even those with higher rates of known risk factors—further attest to these benefits. Moreover, low-risk patients can be readily identified and classified. Specifically, CONREP may be a more appropriate setting than continued inpatient hospitalization for older patients with severe mental illness, particularly those with more extended hospital stays, those who belong to the NGI commitment category, and those who have psychotic disorder diagnoses without co-occurring personality or substance use disorders. Such placement is consistent with RNR principles in managing low-risk patients.

## Conclusions

### Conclusions from the 2024 California DSH CONREP effectiveness report

The findings support the expansion of CONREP services—a California effort begun in 2021. The data suggest that broadening program access could reduce recidivism even among higher-risk populations. Although the directly discharged group had more risk factors, these did not account for the dramatically higher recidivism rates; logistic regression revealed that direct discharge was the most impactful predictor of rearrest. Furthermore, the model correctly classified 83% of the non-recidivists, suggesting that low-risk patients can be accurately identified and better served in the community. Expanding CONREP would allow more patients access to community reintegration programs and support, enabling treatment in the least restrictive environment and consistent with the RNR principles.

Several limitations should be acknowledged, including the reliance on arrest data as the primary outcome measure, potential data inconsistencies and inaccuracies, limited analysis of variable interactions, and incomplete death record access. Nevertheless, our conclusions are based on a substantial sample (2613 patients) over a meaningful period (2012–2017, with follow-up through 2018), and the differences between directly discharged and CONREP-treated patients support firm conclusions about the program’s effectiveness.

Differences in rearrest rates compared with the McDermott et al. study^1^ may be attributed to differences in time frames, means of controlling for time effects, population differences, data sources (eg, Google search for convictions vs. DOJ data), and legislative changes, such as California’s Proposition 47 in 2014. In contrast to McDermott et al.’s findings, our study found that the effect of CONREP treatment persisted after program completion. This study also included forensic patients with multiple commitment types.

This comprehensive evaluation adds to the growing body of evidence supporting supervised outpatient treatment as an effective approach for managing forensic patients in the community while maintaining public safety and promoting successful rehabilitation.

### Conclusions from effectiveness of supervised community release programs for forensic patients

Compulsory community treatment of discharged forensic hospital patients does indeed work. These recent findings are congruent with decades of earlier research and show that forensic patients with psychotic illnesses and lower criminality can be safely and effectively treated in the community. CONREP, with its guardrails and incentives, provides an off-ramp from carceral settings, allowing safe reintegration and solidifying treatment gains. Recent studies confirm that Compulsory community treatment substantially reduces further justice involvement for forensic patients following their release from forensic hospitals. Despite the challenge of drawing generalizable conclusions from studies with varying outcome measures (eg, reconviction vs. rearrest vs. rehospitalization), patient characteristics, commitment schemes, follow-up durations, treatment protocols, comparison group types, and legal/social contexts, the overall evidence demonstrates that risk factors for recidivism—such as lack of community treatment, substance use disorders, personality disorders, male gender, and younger age with higher index offense severity (an inverse relationship to recidivism per Laskorunsky^18^)—are significantly mitigated by supervised community treatment programs.

The empirical evidence demonstrates that such programs significantly reduce recidivism among forensic patients following psychiatric hospitalization, with the California CONREP study showing a fourfold to sevenfold reduction. This effect is consistent across diverse jurisdictions and legal frameworks. Longer treatment durations correlate with better outcomes, although the relationship between initial hospital stay and recidivism is complex. Moreover, racial disparities in forensic mental health systems underscore structural barriers and potential biases that warrant further examination.

Overall, the data provide compelling evidence that adequately structured, Compulsory community treatment is safe and effective. Reduced recidivism via CONREP and other court-supervised programs enhances patient autonomy and functioning and improves public safety. Future program development should focus on expanding access, addressing systemic disparities, and ensuring adequate resources for sustained implementation.
